# Evaluating the Implementation of the GREAT4Diabetes WhatsApp Chatbot to Educate People With Type 2 Diabetes During the COVID-19 Pandemic: Convergent Mixed Methods Study

**DOI:** 10.2196/37882

**Published:** 2022-06-24

**Authors:** Robert Mash, Darcelle Schouw, Alex Emilio Fischer

**Affiliations:** 1 Faculty of Medicine and Health Sciences Stellenbosch University Cape Town South Africa; 2 Aviro Health Cape Town South Africa

**Keywords:** COVID-19, diabetes, primary care, patient education and counseling, mobile health, mHealth, eHealth, telemedicine, South Africa, mobile phone

## Abstract

**Background:**

In South Africa, diabetes is a leading cause of morbidity and mortality, which was exacerbated during the COVID-19 pandemic. Most education and counseling activities were stopped during the lockdown, and the GREAT4Diabetes WhatsApp Chatbot was innovated to fill this gap.

**Objective:**

This study aimed to evaluate the implementation of the chatbot in Cape Town, South Africa, between May and October 2021.

**Methods:**

Convergent mixed methods were used to evaluate the implementation outcomes: acceptability, adoption, appropriateness, feasibility, fidelity, cost, coverage, effects, and sustainability. Quantitative data were derived from the chatbot and analyzed using the SPSS. Qualitative data were collected from key informants and analyzed using the framework method assisted by Atlas-ti. The chatbot provided users with 16 voice messages and graphics in English, Afrikaans, or Xhosa. Messages focused on COVID-19 infection and self-management of type 2 diabetes.

**Results:**

The chatbot was adopted by the Metro Health Services to assist people with diabetes who had restricted health care during the lockdown and were at a higher risk of hospitalization and death from COVID-19 infection. The chatbot was disseminated via health care workers in primary care facilities and local nonprofit organizations and via local media and television. Two technical glitches interrupted the dissemination but did not substantially affect user behavior. Minor changes were made to the chatbot to improve its utility. Many patients had access to smartphones and were able to use the chatbot via WhatsApp. Overall, 8158 people connected with the chatbot and 4577 (56.1%) proceeded to listen to the messages, with 12.56% (575/4577) of them listening to all 16 messages, mostly within 32 days. The incremental setup costs were ZAR 255,000 (US $16,876) and operational costs over 6 months were ZAR 462,473 (US $30,607). More than 90% of the users who listened to each message found them useful. Of the 533 who completed the whole program, 351 (71.1%) said they changed their self-management a lot and 87.6% (369/421) were more confident. Most users changed their lifestyles in terms of diet (315/414, 76.1%) and physical activity (222/414, 53.6%). Health care workers also saw benefits to patients and recommended that the service continues. Sustainability of the chatbot will depend on the future policy of the provincial Department of Health toward mobile health and the willingness to contract with Aviro Health. There is the potential to go to scale and include other languages and chronic conditions.

**Conclusions:**

The chatbot shows great potential to complement traditional health care approaches for people with diabetes and assist with more comprehensive patient education. Further research is needed to fully explore the patient’s experience of the chatbot and evaluate its effectiveness in our context.

## Introduction

### Background

Diabetes is the leading cause of death in women in South Africa and the second overall cause of death after tuberculosis [1]. One in four South Africans aged >45 years have diabetes, and in Cape Town, even higher prevalence rates have been reported [2]. There are approximately 100,000 people with diabetes in the Cape Town Metro Health Services (MHS) database [3].

During the COVID-19 pandemic, the de-escalation of facility-based primary care meant that people attended the facilities less often and many received their medication via home delivery [4]. Support groups and group empowerment were mostly stopped, and individual patient education and counseling were much less frequent. At the same time, people with diabetes were among those most at risk of hospitalization and death from COVID-19 infection, especially if they had poorly controlled diabetes [5,6].

Therefore, alternative mechanisms were necessary to improve patient education, self-management, and levels of glycemic control, while maintaining physical distance and de-escalation of services. In South Africa, cell phone coverage is estimated at 82% of the population and extends to all socioeconomic groups [7]. The South African National Department of Health has promoted strategies to improve health through mobile health (mHealth) technology [8]. So far, most initiatives have focused on maternal health and HIV, with very few targeting noncommunicable diseases [9,10].

A review of systematic reviews on the effectiveness of mHealth interventions on diabetes and obesity treatment concluded that mHealth is a useful tool that can reduce the hemoglobin A_1c_ (HbA_1c_; −0.3% to −0.5%) level and weight (−1.0 to −2.4 kg) [11]. A systematic review and meta-analysis of 24 clinical trials also concluded that tailored mobile educational messages can improve HbA_1c_ levels in patients with type 2 diabetes [12].

A few studies have been conducted earlier on WhatsApp messaging and type 2 diabetes. In South Africa, people with diabetes said that WhatsApp was their preferred technology and wanted education to focus on diet, nutrition, and physical activity [13]. Clinical trials in the United States, Brazil, and Saudi Arabia have shown that WhatsApp education programs can be effective in improving knowledge, self-efficacy, adherence, and glycemic control (a reduction of approximately 0.6% in the HbA_1c_ level) [14-16]. WhatsApp education can also be as effective as group education programs [17]. However, little research has investigated WhatsApp messaging for diabetes in low- and middle-income countries.

The Division of Family Medicine and Primary Care at Stellenbosch University, in partnership with the MHS and Aviro Health, designed a project to provide patient education to people with type 2 diabetes in the MHS via audio messages in WhatsApp during the COVID-19 pandemic. During the crisis, this approach to disseminating messages was shown to be successful in other sectors, such as education and religion, where daily church services were sent to poor communities using WhatsApp audio files [18-20].

### Objectives

The aim of this study was to evaluate the implementation of the GREAT4Diabetes WhatsApp Chatbot education program in the MHS. This study focused on a range of implementation outcomes: adoption, appropriateness, acceptability, feasibility, fidelity, coverage, cost, effects, and sustainability of the initiative.

## Methods

### Study Design

A convergent mixed methods study combined quantitative and qualitative data to evaluate the implementation outcomes (Textbox 1). The evaluation focused on the initial implementation of the chatbot in the Northern Tygerberg Substructure (NTSS) over a 6-month period from May to October 2021.

**Description of the implementation outcomes.**

**Implementation outcome and description**

- Acceptability: Why did stakeholders perceive that it was worth doing? What were the factors for and against this?
- Adoption: Why did stakeholders decide to adopt the intervention? What were the key factors they considered in making this decision?
- Appropriateness: Did stakeholders perceive that the intervention was fit for purpose?
- Feasibility: How feasible was it to implement successfully? What were the factors that enabled and hindered implementation?
- Fidelity: How was the intervention modified or customized to make it work? Why was this necessary?
- Coverage: How many people were reached and who were they?
- Cost: What were the incremental setup and operational costs?
- Effects: What was the effect on people’s self-management?
- Sustainability: Should this be sustained? What are the future opportunities and threats to the sustainability of the intervention? Can implementation be taken to scale?

### Setting

The MHS served the uninsured population of Cape Town who were dependent on the public sector. The population of the NTSS was estimated at 1,081,292 in 2019, and 78% to 90% of the population were uninsured and dependent on the public sector [21]. The leading causes of premature death were interpersonal violence, HIV or AIDS, ischemic heart disease, tuberculosis, stroke, road injuries, diabetes, and lung cancer. Health services in the NTSS were provided by 3 community health centers (open 24 hours) and 11 community day centers (open office hours). These primary care facilities included medical officers, nurse practitioners, and other members of a multidisciplinary team, and each community health center had a family physician (specialist in family medicine). Community-based services were offered via nonprofit organizations under contracts with the MHS. They employed teams of community health workers (CHWs) coordinated by professional nurses and responsible for designated communities (1 CHW for approximately 250 households). The CHWs would visit all the households that they were responsible for on a regular basis. The CHW teams were also connected to a specific primary care facility to form a larger primary health care team comprising facility-based and community-based health workers.

### Design of the Intervention

**Figure 1:**
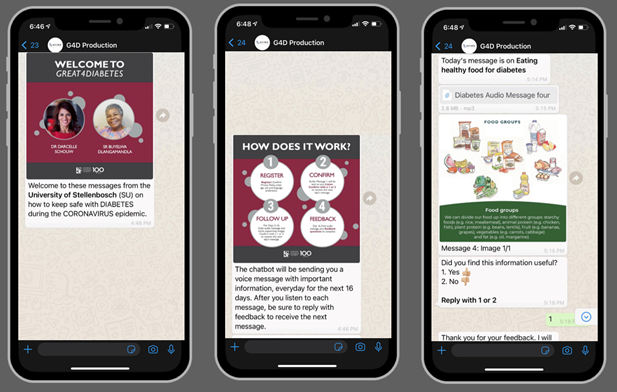
Screenshots of the GREAT4Diabetes WhatsApp Chatbot.

The intervention consisted of 16 three- to four-minute audio messages, which were sometimes supported by a picture (Figure 1). Once a person with diabetes sent the message *Hi* to the designated WhatsApp number, they registered for the program, accepted the standard terms and conditions of Aviro Health and shared key information (age, gender, and language preference). They then automatically received the first audio message. After each message, they had to reply to a question (whether the message was useful) to receive the next message. They could stop receiving messages at any time and were asked to provide a reason for stopping. After the last message, they were asked several questions about self-management of diabetes (Did they change their behavior? What behavior did they change? Did their confidence improve?) and were also able to give free-text feedback.

The content of the audio messages was derived from the Group Empowerment and Training (GREAT) program [22], which was rolled out nationally before the COVID-19 pandemic. The audio messages were recorded in English, Afrikaans, and Xhosa, in a professional recording studio, by members of the GREAT team from Stellenbosch University. Aviro Health was responsible for the WhatsApp Chatbot technology.

Aviro Health set up a content management system and a flow builder that organized the use of this content in a particular sequence and according to specific rules. Aviro Health then sent the messages to a WhatsApp interface provider, in which they entered a queue for distribution and were sent out. A database recorded all the events within the system and what happened with the messages. An extraction mechanism sifted through the raw data to highlight and report on key activities. Once developed, the chatbot was tested using a temporary cell phone number by team members, selected patients with diabetes, and MHS management. The total amount of data required to download all the messages was 94 MB.

### Collection and Analysis of Quantitative Data

The data automatically captured by Aviro Health provided information on *coverage*: the number of people accessing the service and their age, gender, and language preferences. In addition, the number landing on the chatbot, accepting the terms and conditions, listening to each message, and stopping could be determined.

The chatbot also captured data on the reported *effects* of the program. Data were collected on whether participants found the messages useful, as well as on changes in their confidence to self-manage their diabetes. They were specifically asked about changes in behavior with regard to diet, physical activity, adherence to treatment, tobacco smoking, alcohol intake, and foot care.

Stellenbosch University also collected data on the incremental setup and operational *costs* they experienced over the 6-month period.

Data captured by the chatbot were exported into an Excel spreadsheet and then imported into SPSS (version 27; IBM Corp) for analysis. The analysis was mostly descriptive, with categorical data presented as frequencies and percentages and numerical data as means and SDs.

Inferential analysis was conducted to examine any relationships between demographic data and changes in behavior or completion of the program. Categorical variables were compared using the chi-square test, whereas numerical and categorical variables were compared using either the independent samples 2-tailed *t* test or ANOVA, depending on the number of categories.

### Collection and Analysis of Qualitative Data

Descriptive exploratory semistructured interviews with key informants were conducted to explore the acceptability, appropriateness, adoption, feasibility, fidelity, and sustainability of the intervention. Key informants were purposively selected, as shown in Table 1, from Stellenbosch University, Aviro Health, MHS management, and health care workers. It was not possible to conduct face-to-face interviews with patients during the COVID-19 pandemic, and their contact details were protected by the Protection of Personal Information Act and could not be shared. Some patients gave qualitative feedback on the chatbot after the last message, and some quotes were selected to support the quantitative findings on the effects of the chatbot.

Interviews were conducted in English by the 2 authors (DS and RM) face to face, via the internet, or telephonically and lasted between 30 and 60 minutes. All interviews were recorded. An interview guide was used to ensure that all the implementation outcomes were explored. In some cases, a small group interview was conducted when people were available at the same time.

Audiotapes were transcribed verbatim and checked for errors or omissions. Transcripts were analyzed inductively to identify themes related to the implementation outcomes. Atlas.ti software was used to assist with the analysis. The first author, DS, performed most of the analyses, whereas the second author confirmed the coding index and interpretation of data. The researchers used the framework method to analyze the data [23]:

1. Familiarization: reading the transcripts and identifying key issues that need to be coded
2. Coding index: creating an index of codes and organizing them in categories
3. Coding: coding all data according to the coding index
4. Charting: gathering all data on the same code or category together in a chart
5. Interpretation: interpreting each chart for the key themes, range of ideas and experiences within a theme, and any relationships among themes

The different data sources and perspectives were triangulated in the analysis, which contributed to its credibility and trustworthiness. In terms of reflexivity and confirmability, DS is a biokineticist by background and employed as a researcher at Stellenbosch University. She recorded both the English and Afrikaans versions of the messages in the chatbot and acted as the project coordinator. She had prior experience in qualitative interviewing. RM is a family physician and an established researcher at Stellenbosch University with experience in qualitative research. He had previously led the GREAT for Diabetes project on which the voice messages were based. Both researchers were involved in the implementation and evaluation of the project. Guidelines for reporting of mHealth interventions were also followed [24].

**Table 1:** Characteristics of key informants (N=23).

Categories	Participants	Interviews, n (%)
MHS^a^ managers	Chief director and director of NTSS^b^	2 (9)
Primary care facilities	A total of 2 family physicians, 2 medical officers, 2 professional nurses, 1 facility manager, and 1 health educator	8 (35)
Nonprofit organization 1	In all, 1 program manager, 1 professional nurse, and 2 CHWs^c^	4 (17)
Nonprofit organization 2	In all, 1 program manager, 1 professional nurse, 2 CHWs, and 1 dietician	5 (22)
Aviro Health	Chief executive officer, production manager, and implementation manager	3 (13)
Stellenbosch University	Project coordinator	1 (4)

^a^MHS: Metro Health Services.

^b^NTSS: Northern Tygerberg Substructure.

^c^CHW: community health worker.

### Ethics Approval

The study was approved by the Health Research Ethics Committee at Stellenbosch University (reference 21/03/006-COVID-19), and permission was obtained from MHS and Aviro Health.

## Results

This section integrates the findings derived from both quantitative and qualitative data and presents them according to the implementation outcomes.

### Acceptability, Adoption, and Appropriateness

The motivation to adopt the GREAT4Diabetes WhatsApp Chatbot was to improve patient education and levels of glycemic control, while maintaining physical distance and de-escalation of services during the COVID-19 pandemic:

COVID prompted us to think differently in many areas. And I think the whole telemedicine, using technology to reach your target beneficiary, has become important...value of self-management in the client, assisted self-management or supported self-management. And I viewed this is the way to support your uneducated and educated clients in managing their disease.

MHS manager

De-escalation of services was vital to free up the capacity to handle the surge in patients with COVID-19 infection and allow facilities to maintain adequate social distancing. At the same time, it was dangerous for people with diabetes to congregate at health centers and travel via public transport. During the COVID-19 pandemic, services at primary health care facilities were reorganized, which meant that people attended the facilities less often, support groups were stopped, and individual patient education and counseling were infrequent. People with diabetes were among those at the highest risk of hospitalization and death from COVID-19 infection, especially if they had poorly controlled diabetes. The chatbot could therefore provide self-management support to people with diabetes, while keeping them safe and avoiding congestion at primary care facilities. Therefore, the MHS management accepted and adopted this motivation.

The MHS had an existing relationship with Aviro Health and Chatbot technology through another COVID-19–related initiative called the Pocket Clinic, which was designed to help patients request home delivery of medication and update their address on the system. This paved the way for the adoption of the chatbot:

It’s the ability to piggyback a prevention and promotion message onto an existing electronic platform and target people who would benefit the most from it.

MHS manager

Likewise, Aviro Health had prior experience of developing WhatsApp-based products to interact with and educate patients, which made it easy for them to adopt the idea for a chatbot. Aviro Health was particularly keen to include diabetes, as it had previously mostly focused on infectious diseases. The proposed chatbot was simpler in design than other products, as it implemented existing content instead of using the traditional in-house analysis and design process to respond urgently to the crisis. The content was derived from the Living GREAT with Diabetes program, which was viewed as an appropriate group empowerment approach for the same target audience.

### Feasibility and Fidelity

#### Recruitment of Users and Technical Challenges

Users were initially recruited from the NTSS. Pamphlets and posters were made available to the health centers and nonprofit organizations. Patients were introduced to the chatbot by family physicians, medical officers, professional nurses, and health promoters in the facilities, as well as by professional nurses, CHWs, and dieticians in the community. Initially, the uptake was low, and a roadshow was organized throughout the substructure to introduce the chatbot and explain how it worked.

This roadshow was necessary to raise awareness among and motivate health care workers who were already overburdened with the challenges of reorganizing primary health care and responding to the COVID-19 pandemic. Health care workers might see promoting the chatbot as an additional task they were asked to do:

Staff morale was also low at some points in Covid, so to get people motivated to do an additional task is a problem. Because we’re trying to...we had an integrated service. So in your room you’re meant to be able to see to all the primary illness, you’re meant to be doing HIV testing in your room, you’re meant to be doing other screening in your room, so now you’ve got an additional thing to do which becomes a problem.

Family physician

CHWs received face-to-face training on how to use the chatbot and share it with patients during home visits. It was important to demonstrate the chatbot and engage them in the value it could add during the pandemic, especially to those with poorly controlled diabetes:

And yes, also in my experience, I found that it wasn’t going to work, just to come in, share the programme with them and ask them to do it. I had to get their buy- I had to motivate the community health workers to get their buy-in and also to focus on them and to see how they’re doing with and collaborating with them asking their input, how would it work best?

Stellenbosch University

However, several medical officers and professional nurses experienced the chatbot as a great initiative that could be used as part of their normal consultations and reported saving time following its introduction. The chatbot was a valuable tool to complement the education of patients and could be quickly explained to the patient. In addition, illiterate patients, who might struggle with written information, could easily listen to messages:

So I sort of incorporated into, into my consultations here. When I was working at the, at the sort of outpatient department. I was speaking to most of, all my diabetics, in fact, I’ve been sort of advocating, because normally you would educate patients, give them advice on the medications or diet and so on, but I feel it’s quite helpful, because then you don’t have to speak so much.

Medical officer

In facilities that were short staffed and used several different locums, it was difficult to continuously orientate new clinicians to the chatbot. However, other health care workers, such as health promotion officers, could also successfully inform patients about the service:

I said with the locum staff now every time there’s a different locum working now you got to explain it to them. So somehow the process then falls flat.

Family physician

Following this, the number of users started to increase but also coincided with the first technical problem. Over a 3-week period, 183 users received their first message repeatedly. As each message started in a similar manner, it was assumed that users did not realize the messages were different. Eventually, the technical problem was recognized and corrected when management at the MHS confirmed it. Despite the technical glitch, users who received duplicates during this period did not show any significant difference in drop-off compared with users who received the correct flow. All users who experienced these issues were identified and sent a message notifying them of the error and inviting them to continue using the service. Health care workers, however, lost confidence in promoting the service when they were aware of the technical problem. Aviro Health sent messages to users via WhatsApp to recommence their journey. In addition, Stellenbosch University informed the health workers when the chatbot was functioning again and asked them to inform patients:

So she did ask me what’s happening, the messages had stopped. So I said to her that I will get back to her. I will get feedback from the office.

CHW

In an attempt to drive more users to the chatbot, Stellenbosch University then issued a press release to inform the media about the service and communicate directly to the public. The initial pilot was intended for only 500 completed journeys at NTSS, but a series of local radio interviews culminated in a news story on national television. This strategy was successful in increasing the number of users, and the news story attracted more than 6000 users in a day. However, this volume stressed the chatbot, which was designed to host only a few dozen journeys a day. This stress blocked the dissemination of more than 1000 messages to users, as messages were queued up in the system and eventually dropped as there were too many messages at once. Aviro Health was able to quickly redesign the back end to accommodate much higher volumes than anticipated, retime, and batch messages, thus allowing them through the system:

Yes it was a TV news station on SABC or whatever, and then we landed our second glitch. So we had the numbers up. What Aviro did not let us know was that they only had, they did not have capacity for that amount of people. What we had communicated with them prior was to say that before every interview, before every radio interview, before every television interview, we would inform them, which we did.

Stellenbosch University

Recruitment was also facilitated by word of mouth from patients, health care workers, and even MHS managers who told friends and relatives about the chatbot:

I’ve got a sister-in-law who’s diabetic and who developed Covid and she used it as well...well actually I’m just remembering my brother-in-law who stays in Gauteng also used it.

MHS manager

In some facilities, health promoters played voice messages to patients as part of individual or group education sessions held in the facility. Similarly, in the community, patients were introduced to the chatbot during support or adherence group meetings:

Because of Covid and the reason that we can’t get people into the Day Hospital. All of our rooms...consulting rooms are all occupied. So we have a little space in the garden where there’s an under-roof where we go and sit at times but it’s not conducive because if one come in, everybody wants to come in.

Health promoter

#### Patients’ Readiness to Use Technology

Most health workers and patients found the process easy and self-explanatory and were able to follow the instructions to save the number on their phones and send a message on WhatsApp to receive the first voice message.

I think that people are ready for this type of technology especially from the facilities. Everything is moving towards technology. Everything is moving...the only thing that I really think would be a threat is where people don’t have access to internet.

Medical officer

Patients who did not understand how to use the chatbot received assistance from the CHWs or family members. For example, some older people thought they had to dial the number instead of sending a message on WhatsApp or struggled with saving the number as a contact on their phone:

I just had to sit next to them and link them up one by one. When they were here with their phones, I had to sit next to them

Health promoter

It was noted that older people more often made use of analog phones, which could not use WhatsApp, whereas most of the younger people had smartphones. When patients did not have a smartphone, some CHWs used their own phones and made repeat visits for them to listen to voice messages. Others requested assistance from family members with a smartphone. However, these solutions were not always feasible. Health workers at the primary health care facilities and nonprofit organizations in the community also held group sessions where they shared messages as part of chronic disease education:

A lot of the older people, they didn’t have smart phones like they would have a phone but it wouldn’t be a smart phone. It would be just like a normal analogue phone and data was an issue for some of the patients. That was the only two factors. For the people that had a smart phone that were cell phone illiterate, they usually had a family member or earlier on you know that could help them with it.

Medical officer

Data issues were not a major challenge to accessing the chatbot, as most people had already made use of WhatsApp calls and had data for this purpose. For those who struggled to afford data, there were other options. All facilities were equipped with free open-access Wi-Fi, and the City of Cape Town had installed additional routers in the NTSS, which meant that anybody in close proximity could access the chatbot. Patients who had gone for consultation and were introduced to the service at the facilities could retrieve the first voice message while waiting for their medication. They could also access the facility on days when they did not have appointments and make use of the free Wi-Fi. Alternative options include accessing data through free Wi-Fi at shops and malls in the local community:

No it’s not so much that they don’t have data. Because they won’t have a piece of bread in the house but data they will have on their phone. Those who have got smart phones you understand.

CHW

#### Modification of the Chatbot

The content of the messages was not changed during the study; however, modifications were made to the interface to improve the flow and retention of users. Explanatory text was simplified, particularly the initial consent and acceptance of terms and conditions. The text at the landing and consent stages was replaced by infographics that explained how to use the chatbot. In addition, an infographic introduced photographs of the 2 presenters to make a more personal connection with the voices:

Based on user feedback and analysis of drop-offs through the flow, Aviro modified the content (simplified that wording, reduced the consent, added more emoticons, and made slight changes to the flow) to make it easier for those with diabetes to navigate and understand the experience.

Aviro Health

### Coverage

**Figure 2:**
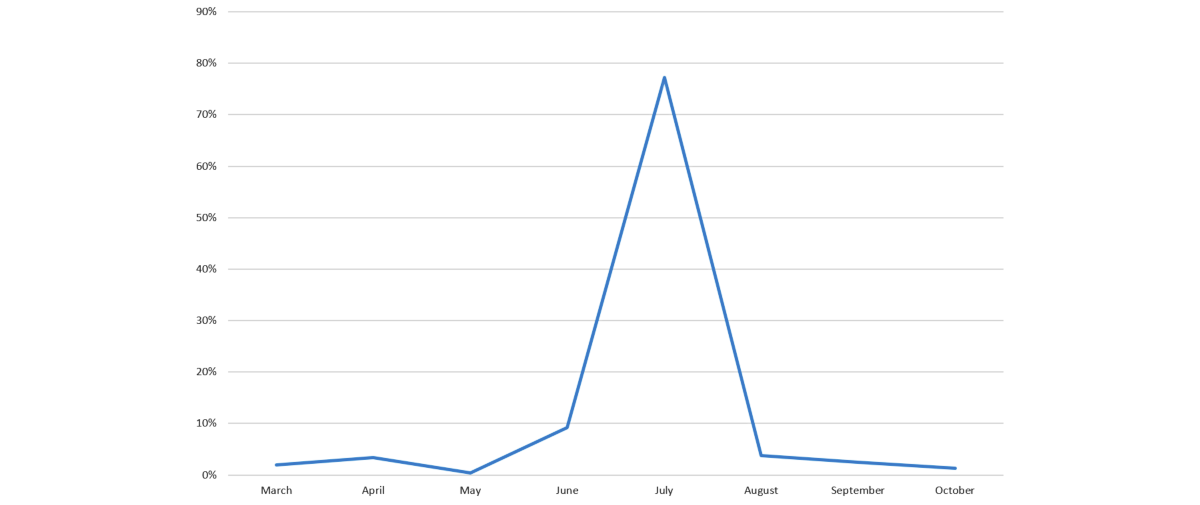
Percentage of people landing on the chatbot per month in 2021 (N=4716).

Between March 2021 and October 2021, a total of 8158 people landed on the chatbot. However, of 8158 people, only 4716 (57.83%) responded to the terms and conditions, and their distribution per month is shown in Figure 2. Overall, 77.29% (3645/4716) of these people connected during July 2021, which coincided with the most intense period of media exposure, including television.

Of 8158 who considered the terms and conditions, 4577 (97.1%) agreed to them but 139 (2.9%) did not. Overall, 81.01% (3708/4577) provided demographic information and the mean age of the participants was 51.0 (SD 12.4) years.

Out of the 4577 participants who provided demographic details, 2066 (55.7%) were women, 1632 (44%) were men, and 10 (0.3%) were identified as other. Most preferred English (2281/4577, 61.5%), but 34.9% (1293/4577) chose Afrikaans and 3.58% (134/4577) chose Xhosa.

Of the 4577 participants, 263 (5.7%) requested to stop the chatbot, and 164 (62.4%) of them did so within the first 24 hours. Of those who stopped, 78.3% (206/263) gave feedback and 11.7% (24/263) said it was not useful, 46.1% (95/263) said it was because of data issues, and 42.2% (87/263) did not give a specific reason. Table 2 presents the proportion of participants who started receiving each message. Only 12.56% (575/4577) of those who received message 1 also received the final message 16. Although the television interviews drew the largest number of new users, only 4.53% (280/6173) of these users completed the program, whereas 12% (53/441) of the users from radio and 16.47% (28/170) of the users recruited by health care workers completed the program.

Table 3 shows the time taken to complete the entire program. Of 510 participants, only 122 (23.9%) participants completed within the expected 16 days, although most of them completed within 32 days (n=362, 71%).

Patients also reported forwarding the messages to others, which increased the coverage:

Thank you for the information, very helpful. I did forward your messages to my friends and families who are diabetic.

Female, 49 years

**Table 2:** Proportion of participants receiving each message (N=4577).

Message number and topic	Participants, n (%)
Message 1: Avoiding COVID-19 infection	4577 (100)
Message 2: Reducing COVID-19 infection	3293 (71.93)
Message 3: What is diabetes?	2534 (55.42)
Message 4: Eating healthy food types	2173 (47.48)
Message 5: Portion sizes	1910 (41.74)
Message 6: Cooking and meals	1650 (36)
Message 7: Drinks	1470 (32.12)
Message 8: Aerobic activity	1210 (26.39)
Message 9: Resistance	998 (21.83)
Message 10: Medication	896 (19.56)
Message 11: Low blood sugar	837 (18.34)
Message 12: High blood sugar	798 (17.42)
Message 13: Mental health	752 (16.43)
Message 14: Control and complications	697 (15.17)
Message 15: Feet	652 (14.23)
Message 16: Visiting the clinic	575 (12.61)

**Table 3:** Time taken to complete the program (N=510).

Within number of days	Participants, n (%)
16	122 (23.9)
32	240 (47.1)
48	69 (13.5)
64	56 (11)
80	14 (2.7)
96	3 (0.6)
112	5 (1)
128	1 (0)

### Costs

Table 4 lists the incremental setup and operational costs involved in the chatbot. Over the 6-month period, it cost ZAR 255,000 (US $16,876) for setup and ZAR 462,473 (US $30,607) in implementation and operational costs. The operational cost for each person who accessed the chatbot was, therefore, US $6.69. This amount would decrease if the number of users increased, as operational costs were not related to the number of users. The monthly operational cost was US $5101.

**Table 4:** Incremental setup and operational costs for 6 months.

Type of costs			Costs (ZAR)		Costs (US $)^a^	
**Setup**						
	Design and development of Chatbot	245,000				16,214
	Recording studio	10,000				662
	Total	255,000				16,876
**Operational**						
	Project coordinator (over 6 months)	150,000		9927		
	Chatbot operations	291,000		19,259		
	Promotional materials, design, and printing	21,473		1421		
	Total	462,473		30,607		

^a^1$=ZAR15.11 on March 10, 2022.

### Effects

Table 5 shows the proportion of participants who listened to each message, provided feedback, and found it useful. More than 90% of those who listened to the messages found them to be useful. The least useful message was the first one on the COVID-19 infection (3093/3293, 93.92%), and the most useful was mental health and stress (691/697, 99.1%).

Overall, 494 participants gave feedback on changes in their behavior at the end of the program; of these, 351 (71.1%) reported that they changed *a lot*, whereas 123 (24.9%) reported that they changed *a little*, and 20 (4%) reported no change:

I did find the messages very inspiring and to put it in action is very important for my health.

Female, 70 years

All the sessions was applicable to me, I gain more information at large on how to look and care about myself. I was negative about my diabetes, after going through with you all these sessions, I am positive about it and would like you to continue the excellent work, thanks a lot. Regards.

Male, 52 years

In addition, of the 421 participants, 369 (87.6%) reported that they were much more confident in self-care for their diabetes:

You help me a lot about a knowledge and confidence thanks a lot.

Male, 39 years

Table 6 presents the behaviors that people reported as changing because of the program. The most common changes were in diet and physical activity. A substantial proportion also improved their adherence to medication, foot care, and stress management. Only a small proportion changed their alcohol use or tobacco smoking, although the proportion of smokers or using alcohol was unknown:

Yes thank you very much, now I understand my diabetes condition a lot and already feeling much better I use to have rash and itchy, now it’s gone, I changed the way I eat.

Male, 54 years

Wish there was more on exercise.

Female, 49 years

I can say I’ve learnt a lot about diabetes and can educate my community more about how to manage and live healthy lifestyle with diabetes.

Female, 49 years

There was no significant association between age, gender, or language preference and the probability of changing behavior. Those who completed all 16 messages were likely to be slightly older (mean 52.0 vs 50.1 years; mean difference 1.18, 95% CI 0.043-2.32; P=.04). There was no association between gender or language preference and those who completed the program.

**Table 5:** Proportion of participants who found each message useful.

Message number and topic	Participants, N	Participants who found it useful, n (%)
Message 1: Avoiding the COVID-19 infection	3293	3093 (93.9)
Message 2: Reducing the COVID-19 infection	2534	2382 (94)
Message 3: What is diabetes?	2173	2099 (96.6)
Message 4: Eating healthy food types	1910	1835 (96.6)
Message 5: Portion sizes	1650	1660 (97.1)
Message 6: Cooking and meals	1470	1397 (95)
Message 7: Drinks	1210	1144 (94.5)
Message 8: Aerobic activity	998	948 (95)
Message 9: Resistance	896	877 (97.9)
Message 10: Medication	—^a^	—
Message 11: Low blood sugar	798	775 (97.1)
Message 12: High blood sugar	752	731 (97.2)
Message 13: Mental health	697	691 (99.1)
Message 14: Control and complications	652	635 (97.4)
Message 15: Feet	575	565 (98.3)
Message 16: Visiting the clinic	533	524 (98.3)

^a^Data not available.

**Table 6:** Changes in specific behavioral issues in self-care for diabetes (N=414).

Behavioral change	Yes, n (%)
I changed my diet	315 (76.1)
I changed physical activity	222 (53.6)
I improved adherence to medication	182 (44)
I changed my foot care	178 (43)
I changed my stress management	186 (44.9)
I changed my smoking	34 (8.2)
I changed my alcohol intake	46 (11.1)
I changed something else	27 (6.5)

### Sustainability

All respondents agreed that the chatbot service should continue and made many suggestions to improve or extend the service and its integration into the MHS:

Don’t stop what you’re doing, don’t stop. Digital health is the future, but digital health is a complimentary service to what you are doing. We are not getting to the people prof. We’re not getting to people with diseases like diabetes. It’s going to be the leading killer. You know heart disease, these are controllable but there’s like controllable at a personal level.

Aviro Health

I think that this program should definitely be sustained. I think we’ve seen that there is an appetite for it just simply by the number of users who landed...it’s not just about people who complete a program but people who show interest by just on-boarding. And we’ve seen spikes in those numbers. Particularly when there were roadshows.

Aviro Health

Health care workers and managers suggested that additional services be developed for other chronic diseases, such as hypertension, heart failure, asthma, and chronic obstructive pulmonary disease. The chatbot could complement consultations and make them more efficient while providing more comprehensive information than would be possible in a short consultation. A menu of WhatsApp-based patient education programs for chronic conditions could be provided:

It can happen for different chronic diseases it could really help in consultations to cut it a little bit shorter, it’s quite important and I do think it would be helpful to roll it out or try it for different chronic diseases.

Medical officer

The staff who implemented the chatbot recommended that it be introduced to all auxiliary workers, clinicians, and health care workers treating patients with diabetes and incorporated into all consultations and home visits. The chatbot should be promoted as part of service delivery through the usual management structures:

From the facility perspective, I think having to get all the health workers involved and not just speak to the Doctors, but like I say the CNPs and the CDCs as well, so we are a CHC but there are CDCs in our areas, so having to maybe speak to the CNPs and everyone that is involved with treating diabetic patients.

Medical officer

I think it should be from our department or all the other sub-structures needs to be informed...we need to be trained on this chat group and it should be a natural thing in the Day Hospital where everybody has their number...all our clinicians have their little flyer and it also explain to the person that maybe missed me when I was talking.

Health educator

In addition, respondents suggested that it should be integrated with the Pocket Clinic as one package of WhatsApp-based services for people. This would consolidate services via WhatsApp and build people’s awareness and confidence in such a system. It was also suggested that the chatbot could be promoted via the parcel of medications that stable patients received:

I know the pocket clinic is working quite well and I think if it is integrated it might work better because I really think like stand-alone things like that don’t work. So once its integrated into a system it might actually work better.

Family physician

But also with the medication, it is theoretically possible to say everybody that’s getting diabetic medication through the CDU system, should automatically with their parcel, get a thing saying “please WhatsApp the following number if you’re interested in getting more information.”

MHS management

Ideally, data from the chatbot service should also be integrated with district health information (eg, single patient viewer) so that health care workers could see who has accessed the material. There might also be an option to suggest the chatbot to patients who had poorly controlled diabetes.

The chatbot could be scaled up if a no-cost health data option was made available to users through cellular networks, similar to the no-cost data packages offered to students during the COVID-19 pandemic and lockdown. This would eliminate financial barriers and enable access to all potential users. In addition, a website could be developed in which users could access messages without any subscription or downloading costs.

Introducing other languages into the chatbot would also allow for it to be scaled up nationally, outside the Western Cape. Some minority and migrant groups within Western Cape might also benefit from other languages.

Finally, the technology needs to ensure that it can go to scale and avoid technical problems experienced during the study period. Communication and monitoring of errors between the WhatsApp service provider and Aviro Health must be improved. Aviro Health also suggested that a feedback or help option could be added to the platform so that end users could directly provide feedback. Going to scale might also improve the service, as Aviro Health commented that the WhatsApp service provider regarded this as a small-scale and relatively unimportant initiative:

Mostly around the technical infrastructure. It is...yeah, in terms of their technical infrastructure there are times where there might be errors. So we send out a message from our system. And like I said, they’re the middleman, so it kind of goes through their console, before it goes to users. And sometimes we didn’t receive error messages or error logs and that is due to kind of how their tech stack is set up.

Aviro Health

## Discussion

### Principal Findings

The chatbot was adopted as a useful innovation in response to the COVID-19 pandemic and was rapidly developed and implemented. It was feasible to implement via health services and media and for people with type 2 diabetes to use in our context. Minor changes were made to the chatbot to improve usability and solve technical glitches. Coverage was sufficient for 8158 participants to land on the chatbot, 4577 to consent, and 575 to complete all 16 messages over a 6-month period. Incremental costs were US $16,876 for setup, and operational costs were US $30,607. Patients reported substantial changes in their confidence in self-managing diabetes and behavior change. All stakeholders supported the continuation of the chatbot, although health services must make a final policy decision in the future. There is potential to include other languages and conditions.

### Discussion of Key Findings

The key findings will be discussed in relation to “the framework (abbreviated NASSS) for studying the Non-adoption and Abandonment of technologies by individuals and the challenges to Scale-up, Spread and Sustainability of such technologies in health and care organizations” [25]. The NASSS framework has 7 components: condition, technology, value proposition, adopters, organization or organizations, wider system, and embedding and adaptation over time. These components have been identified through the synthesis of multiple theories of technological implementation and the realization that many innovations do not succeed for a variety of complex reasons.

The focus of the intervention on diabetes was affirmed by respondents as a key strategy during the COVID-19 pandemic. We were not able to determine any improvement in glycemic control among users, but previous studies have suggested a modest but clinically significant improvement in HbA_1c_ levels [14-16]. Similarly, the number of hospitalizations and deaths averted from COVID-19 infections is unknown. Feedback from patients suggested changes in lifestyle modifications, medication adherence, and confidence in self-management, which would be consistent with improved glycemic control. Ideally, a clinical trial should be conducted to confirm that the intervention is effective in improving control over type 2 diabetes.

Overall, the technology appeared to be a good fit with the target audience, who mostly had smartphones, were familiar with WhatsApp, and could afford data. Several strategies were mentioned for people who did not meet these criteria. Cell phone penetration is known to be high in low-socioeconomic communities in South Africa, and WhatsApp has been identified as the preferred mode of communication for people with diabetes [7,13]. Although several other educational apps for diabetes are available on the market, they may target patients in the private sector with more resources and a different lifestyle than those using the public sector. They may also assume a higher data use. Therefore, the chatbot may be more appropriate for communities dependent on the public sector. To resolve problems more quickly, a help desk function would be useful to allow users to report errors and get assistance. If the chatbot is scaled, it will require more robust systems and regular monitoring to work at a sufficient level of stability to be acceptable outside of a pilot setting.

In terms of the value proposition, the value to health services was the possibility of reduced morbidity and mortality from both diabetes and COVID-19 infection while decongesting primary care facilities and freeing up the capacity to respond to the pandemic. The costs per patient were at par with a monthly prescription for diabetes medication. The per capita cost would also decrease with scale-up and increased reach. The impact of the chatbot will also depend on the coverage and going to scale. Reach was amplified by the use of media, particularly television, but this recruitment strategy is not sustainable. Reach via health care workers was on a much smaller scale, and scale-up would require the use of the chatbot to be embedded routinely into clinical encounters.

Value to the technology company was less certain. The mission of Aviro Health “is to help health care workers focus on more complicated cases by providing technology-enabled services that automate workflows, improve access to quality medical information, and provide digitally-enabled counselling services” [26]. The future commitment of Aviro Health is interdependent on the policy and priorities of the Department of Health and whether they are willing to contract in the longer term. Aviro Health are also keen to change the WhatsApp service provider to prevent technological problems and enable scalability. This would require redesigning the product using new software and additional development costs.

The value to patients was clear in terms of the potential to support a comprehensive understanding of their condition and self-management. Such comprehensive patient education is often lacking in primary care services [27,28]. The large drop-off in potential users that land on a new digital product and actually use it is common in this environment. Each message was found to be useful by those who listened to it. However, only a small proportion of patients completed all 16 messages, and retaining users’ attention over a prolonged period is a challenge for such interventions. This also raises the question as to whether the number of messages should be reduced. Further evaluation of the service by users might answer some of these questions.

The cost of data was minimal, even in poor communities, as 100 MB would cost between US $0.66 and US $0.99 for the entire program. The time taken to listen to the messages did not appear to be an issue, and users could stretch the program for more than 16 days. The reach was limited to the Xhosa-speaking population, although it is likely that many first-language Xhosa speakers chose to listen in English. However, overall, the value proposition appeared to be favorable for all stakeholders.

The chatbot was easily adopted by most users who were already familiar with WhatsApp and smartphones. Older people require assistance in understanding the process. Health care workers overcame their initial resistance to adopting a new task, as they realized that the chatbot could save them time and add value for the patient. However, the promotion of the chatbot needs to be embedded into clinical practice, be part of the orientation of new health care workers, and be introduced to everyone who interacts with patients (eg, pharmacy assistants, pharmacists, and dieticians). The ongoing adoption of the chatbot may also depend on the ownership of the initiative by MHS managers from the facility to district level.

During the pandemic, the MHS showed an ability to innovate and adapt rapidly to the situation, as exemplified by their support for the chatbot. Going forward, however, the organization must make critical decisions regarding the use of digital solutions for primary health care. Almost all facets of primary health care need improved electronic information systems, from the need for electronic medical records to mHealth systems for CHWs. Technological and informational decision-making is complex and difficult, and the place of WhatsApp-based services is not yet clear. There is a need for a coherent and integrated digital architecture and policy rather than an eclectic mix of digital innovations and projects, each trying to solve a problem in isolation. There is also a tension between a desire to innovate in-house, to own the technology and control the product, and a need to move quickly with the help of external companies that already have expertise in the area but on whom you become dependent. The future of the chatbot in its current form is threatened by inertia in such complex decision-making, which may not reach a conclusion quickly enough and might decide against the current model. However, respondents were positive about the potential future contribution of WhatsApp-based services.

The COVID-19 pandemic has highlighted the importance and value of digital health solutions. Examples include telehealth, web-based consultations, remote monitoring, and WhatsApp messaging [29]. Funders such as the Bill and Melinda Gates Foundation have recently emphasized their interest in funding digital health solutions for primary health care, and the climate for such innovations is favorable [30]. There are medicolegal concerns with clinicians using WhatsApp to share patients’ medical details and with the collection of personal information by third parties [31]. Medical ethics and professionalism must adapt their principles to guide professionals in the digital age. However, the chatbot did not raise any such concerns.

### Limitations of the Methods

Quantitative data were limited to what was routinely collected by the chatbot, which was not primarily designed to collect research data. For example, we had no data on where patients were located, their clinical history, glycemic control, or risk factors. Although we hoped to interview patients and obtain their qualitative feedback, this was not possible. First, the COVID-19 pandemic made it impossible to conduct face-to-face interviews. Second, the recent Protection of Personal Information Act in South Africa and standard Aviro Health terms and conditions did not allow for the sharing of confidential patient contact details with the researchers. Qualitative data were obtained by interviewing other key stakeholders. Qualitative researchers were involved in implementing the chatbot and evaluating the implementation. On the one hand, this meant that researchers had in-depth insights into the issues raised by interviewees; on the other hand, they were committed to the project’s success and could have been inclined to a more positive interpretation of the data. The blurring of roles between implementation and implementation evaluation is common in embedded research and implementation science [32].

### Recommendations

This evaluation supports the value of the chatbot and the need to sustain it. Although the intervention itself is relatively simple, the complexity of decision-making around digital health solutions in the health services and financially constrained health sectors may prevent any immediate long-term commitment. Should the health services commit to the chatbot in the future, there is potential to add more languages and chronic conditions and evaluate it further. The Diabetes Alliance strongly recommends the use of such services in South Africa [33]. Such chatbots could have the potential to scale up nationally and even within the region. Future research can evaluate users’ experience and feedback on the content and the effectiveness of the education by measuring clinical outcomes such as HbA_1c_.

### Conclusions

The chatbot was seen as an acceptable initiative by the MHS and quickly adopted during the COVID-19 pandemic to assist people with diabetes. The initiative appeared to be appropriate and useful to patients, with reported improvements in confidence and self-management. The chatbot was feasible to implement despite some technical glitches, and most patients had access to smartphones and sufficient data and were able to navigate the system. There was fidelity to the original design, although the text was simplified and more infographics were added to support the usability and retention of users. Coverage by health care workers was slow and amplified dramatically by the use of radio and television media. Costs were relatively modest and would improve with economies of scale. Respondents thought the chatbot should be sustained and saw the potential for adding languages and other conditions. Sustainability, however, is generally dependent on organizational decision-making around policy, costs, and design of digital health solutions. Further research should explore patients’ perspectives and effectiveness of the chatbot.

## Abbreviations

CHW: community health worker
GREAT: Group Empowerment and Training
HbA_1c_: hemoglobin A_1c_
mHealth: mobile health
MHS: Metro Health Services
NASSS: Non-adoption and Abandonment of technologies by individuals and the challenges to Scale-up, Spread and Sustainability
NTSS: Northern Tygerberg Substructure
